# *In vivo* genome‐editing screen identifies tumor suppressor genes that cooperate with *Trp53* loss during mammary tumorigenesis

**DOI:** 10.1002/1878-0261.13179

**Published:** 2022-01-26

**Authors:** Luuk Heitink, James R. Whittle, François Vaillant, Bianca D. Capaldo, Johanna F. Dekkers, Caleb A. Dawson, Michael J. G. Milevskiy, Elliot Surgenor, Minhsuang Tsai, Huei‐Rong Chen, Michael Christie, Yunshun Chen, Gordon K. Smyth, Marco J. Herold, Andreas Strasser, Geoffrey J. Lindeman, Jane E. Visvader

**Affiliations:** ^1^ ACRF Cancer Biology and Stem Cells Division The Walter and Eliza Hall Institute of Medical Research Parkville Australia; ^2^ Department of Medical Biology The University of Melbourne Parkville Australia; ^3^ Department of Medical Oncology Peter MacCallum Cancer Centre Melbourne Australia; ^4^ Princess Máxima Center for Pediatric Oncology Utrecht The Netherlands; ^5^ Immunology Division The Walter and Eliza Hall Institute of Medical Research Parkville Australia; ^6^ Personalised Oncology Division The Walter and Eliza Hall Institute of Medical Research Parkville Australia; ^7^ Department of Pathology The Royal Melbourne Hospital Parkville Australia; ^8^ Bioinformatics Division The Walter and Eliza Hall Institute of Medical Research Parkville Australia; ^9^ School of Mathematics and Statistics The University of Melbourne Parkville Australia; ^10^ Blood Cells and Blood Cancer Division The Walter and Eliza Hall Institute of Medical Research Parkville Australia

**Keywords:** axin1, breast cancer, CRISPR/Cas9, intraductal, mammary organoids, Prkar1a, Trp53

## Abstract

Breast cancer is a heterogeneous disease that comprises multiple histological and molecular subtypes. To gain insight into mutations that drive breast tumorigenesis, we describe a pipeline for the identification and validation of tumor suppressor genes. Based on an *in vivo* genome‐wide CRISPR/Cas9 screen in *Trp53^+/–^* heterozygous mice, we identified tumor suppressor genes that included the scaffold protein *Axin1*, the protein kinase A regulatory subunit gene *Prkar1a*, as well as the proof‐of‐concept genes *Pten*, *Nf1*, and *Trp53* itself. *Ex vivo* editing of primary mammary epithelial organoids was performed to further interrogate the roles of *Axin1* and *Prkar1a*. Increased proliferation and profound changes in mammary organoid morphology were observed for *Axin1/Trp53* and *Prkar1a/Trp53* double mutants compared to *Pten/Trp53* double mutants. Furthermore, direct *in vivo* genome editing via intraductal injection of lentiviruses engineered to express dual short‐guide RNAs revealed that mutagenesis of *Trp53* and either *Prkar1a*, *Axin1*, or *Pten* markedly accelerated tumor development compared to *Trp53*‐only mutants. This proof‐of‐principle study highlights the application of *in vivo* CRISPR/Cas9 editing for uncovering cooperativity between defects in tumor suppressor genes that elicit mammary tumorigenesis.

## Graphical abstract

*TP53* mutations occur frequently in breast cancer. Here, we describe a framework to identify novel tumor suppressor genes that cooperate with loss of *Trp53* in mammary tumorigenesis using an *in vivo* genome‐wide CRISPR/Cas9 screen. Hits were validated through genetic editing of mammary epithelial organoids *in vitro* and direct editing of ductal cells *in vivo* using engineered lentiviruses.

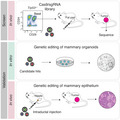

**Abbreviations**
CRISPR: clustered regularly interspaced short palindromic repeats
ER: estrogen receptor
FACS: fluorescence‐activated cell sorting
K: cytokeratin
MMTV: mouse mammary tumor virus
PKA: protein kinase A
PR: progesterone receptor
sgRNA: short‐guide RNA
TCGA: the cancer genome atlas
TMM: trimmed mean of M values
Trp53: tumor protein 53

## 1 Introduction

Breast cancer is a complex disease with clinical and biological heterogeneity that represents a significant challenge for patient management. The different subtypes of breast cancer are thought to reflect the target cell population as well as the specific repertoire of mutations acquired in the preneoplastic phase [1, 2]. Over the past decade, large‐scale genomic studies have highlighted recurrent genetic alterations and numerous mutated genes that occur in the different subtypes of breast cancer [3, 4, 5, 6]. Mapping the early mutagenesis events in mammary epithelial cells is essential for understanding key genomic drivers.

TP53 represents a key tumor suppressor implicated in the protection from breast cancer, with approximately 30% of tumors harboring mutations in *TP53*. The precise nature and functional outcomes of these mutations vary between molecular subtypes [3, 7]. Based on clonal frequency in breast tumors, *TP53* mutations represent a common early event [8]. Notably, breast cancer is a hallmark tumor in patients with Li‐Fraumeni syndrome, ~ 50% of whom carry a germline *TP53* mutation in one allele [9]. This phenotype is recapitulated in the BALB/c‐*Trp53^+/–^* (heterozygous) mouse model, which primarily develops mammary tumors [10]. The long tumor latency implies that additional mutations are required for effective tumorigenesis, one of these being mutation or loss of the wild‐type *Trp53* allele [11].

Genetic screens have emerged as a powerful tool to study biological processes in an unbiased fashion. *In vivo* screens have been carried out in the mammary gland using MMTV‐mediated insertional mutagenesis, leading to the identification of key gain‐of‐function genes in mammary oncogenesis [12, 13]. Conversely, genome‐wide RNA interference (RNAi) screens in mammary epithelial cells *ex vivo* [14, 15, 16] have identified potential candidate tumor suppressors but shRNA knockdown of gene expression is often transient and incomplete. Genome‐wide CRISPR/Cas9 screens present an effective strategy to identify essential genes [17, 18]. Indeed, *in vivo* genome‐wide CRISPR/Cas9 screens have identified mediators of tumorigenesis [19, 20, 21, 22, 23], but these have not yet been explored in mouse models of mammary cancer. Here, we demonstrate the applicability of an *in vivo* genome‐wide CRISPR/Cas9 screen in the haploinsufficient BALB/c‐*Trp53^+/–^* mouse model to identify tumor suppressor genes involved in mammary tumorigenesis. We provide a framework for validation of these loss‐of‐function mutations using genomic editing of primary mammary organoids to assess changes in morphology and proliferation, as well as direct *in vivo* editing of the epithelium via intraductal lentiviral injection to test for tumorigenic capacity.

## 2 Materials and methods

### 2.1 Mice

Wild‐type FVB/N and BALB/c mice were provided by The Walter and Eliza Hall Institute (WEHI) animal facility. MMTV‐Neu (FVB/N), MMTV‐Wnt1 (FVB/N), and BALB/c‐*Trp53^+/–^* were obtained from the Jackson Laboratories. Rosa26‐LSL‐Cas9 mice (kindly provided by D. Hilton) were crossed with FVB/MMTV‐cre (kindly provided by K. Uwe‐Wagner) to obtain FVB/MMTV‐cre/Cas9 mice. All animal experiments conformed to regulatory standards and were approved by the WEHI Animal Ethics Committee (2017.002, 2020.005, 2020.006).

### 2.2 Cell lines and transfections

HEK293T and 3T3 cells were maintained in DMEM (Gibco, Waltham, MA, USA) supplemented with 10% fetal calf serum (FCS). NIH3T3 cells were irradiated with 5000 rads to generate i3T3 cells.

Lentiviral plasmids were transfected into HEK293T cells, and virus was collected 24 h later and concentrated using Amicon Ultra‐15 Centrifugal Filter Units with Ultracel‐199 membrane tubes (Merck, Darmstadt, Germany). Virus titers were defined by titration curves in HEK293T cells.

### 2.3 Plasmids

Plasmid FUCas9Cherry was used to constitutively express Cas9 and plasmid FgH1tUTCyan/mCherry was used for expression of short‐guide RNAs (sgRNAs) [24]. Gblock technology (Integrative DNA technologies) and restriction enzyme cloning were utilized to re‐engineer FgH1tUTCyan or FgH1tUTmCherry to constitutively express two sgRNAs. Single sgRNAs were cloned into FgH1tUTmCherry. Additional plasmids for the mini‐screen were obtained from the Sanger Arrayed Whole Genome Lentiviral CRISPR Library (Sigma, St Louis, MO, USA). sgRNA sequences are listed in Table S1.

### 2.4 *In vivo* CRISPR Screen

Single‐cell suspensions were generated from the 3rd and 4th mammary glands of 12‐ to 15‐week‐old female BALB/c‐*Trp53^+/–^* mice as previously described [25]. Cells were stained with CD29 (HMβ1‐1, Biolegend, San Diego, CA, USA), CD24 (30‐F1, Biolegend), CD45 (30‐F11, BD Bioscience, Franklin Lakes, NJ, USA), CD31 (MEC 13.3, BD Bioscience), and TER‐119 (TER‐119, BD Bioscience) and sorted on a FACS ARIA II (Becton Dickinson, Franklin Lakes, NJ, USA). Basal cells were counted and resuspended with i3T3 cells (2.5 × 10^4^ basal cells per 1 × 10^5^ i3T3 cells) in 6‐well plates in MEC media (DMEM/F12, 10% FCS, Insulin 1 mg·mL^−1^, Hydrocortisone 500 mg·mL^−1^, EGF 100 µg·mL^−1^) in the presence of Y27632 (10 μm). On Day 3, cells were co‐infected with Cas9‐mCherry and the mouse genome‐wide lentiviral library harboring BFP [17] or a vector containing a non‐targeting control short‐guide RNA. Three days after infection, cells were sorted on a FACS ARIA II (Becton Dickinson). Fibroblasts were excluded using staining for CD140b, and epithelial cells positive for mCherry and BFP were isolated by flow cytometry and transplanted (2 × 10^4^ cells) into the cleared mammary fat pads of 3‐ or 4‐week‐old recipient BALB/c mice in the presence of 25% growth factor‐reduced Matrigel (BD Bioscience). Five cohorts of 60 mice were seeded and monitored for tumor development twice weekly. Mice culled from tumor unrelated causes were censored.

### 2.5 Organoid generation and culture

Organoids were cultured as previously described [26]. For organoid proliferation measurements, 1000 cells/10 μL BME were plated in 96‐well plates (Nunc). After 12–14 days, the CellTitre‐Glo Luminescent Cell Viability Assay (Promega, Madison, WI, USA) was used to measure cell viability. Proliferation was determined by dividing the total number of cells after 1 week of culture by the cells seeded initially.

### 2.6 Intraductal mammary injections

FVB/MMTV‐cre/Cas9 (8‐ to 10‐week‐old) mice were anesthetized by administering 10 mg·kg^−1^ xylazine/100 mg·kg^−1^ ketamine by intraperitoneal injection. Mice were placed on their back, and the top part of the nipple of the inguinal gland was sterilized and snipped before insertion of the needle (30G blunt‐ended 10 µL Hamilton syringe) into the primary duct. 10^6^–10^7^ lentiviral transduction units were injected per mammary gland.

### 2.7 Confocal 3D imaging

Organoids were prepared as previously described [27]. For dissociation, organoids were washed with PBS, followed by washing in ice‐cold Cell recovery solution (CORNING, cat. No. 354253) and incubated at 4 °C on a horizontal shaker for 30–60 min. Organoids were then transferred to a 15‐mL tube that had been pre‐coated with 1% BSA and washed with ice‐cold 1% BSA followed by spinning at 70 g for 3 min. The resulting pellet was resuspended in 1 mL 4% paraformaldehyde and incubated for 30 min at 4 °C. Organoids were washed with PBT (PBS, 0.1% Tween) and incubated overnight at 4 °C with primary antibodies against Keratin 5 (rabbit polyclonal, Biolegend, 1/500), E‐cadherin (rat monoclonal antibody, ECCD‐2, 1/250), or Keratin 8/18 (rat monoclonal antibody, TROMA, 1/200), followed by washing and overnight incubation with specific secondary antibodies (listed below), DAPI (4’6‐diamidino‐2‐phenylindole, Thermo Fisher Scientific, 2 μg·mL^−1^), and Alexa Fluor 647 Phalloidin to label F‐actin (Invitrogen, Waltham, MA, USA, 1/100). Secondary antibodies included donkey anti‐rabbit IgG coupled to Alexa Fluor 488 and goat anti‐rat IgG coupled to Alexa Fluor 555 (Invitrogen, 1/400). The following day, organoids were embedded in FUnGI clearing agent [27, 28] before imaging by tiled z‐stacks using a Zeiss LSM 880 or 980 inverted confocal microscope. 3D rendering was performed using Imaris (Bitplane).

### 2.8 Immunohistochemistry

Tumors were collected, fixed in 4% paraformaldehyde, and embedded in paraffin, whereas organoids were resuspended in HistoGel (Epredia) before embedding in paraffin. Sections were subjected to antigen retrieval in sodium citrate pH 6 or target retrieval solution pH 9 (DAKO S2375) at 95 °C for 20 min and incubated with antibodies against K8/18 (TROMAI, DSHB Iowa, 1/600), K5 (PRB160, Biolegend, 1/10000), Ki67 (D3B5, Cell Signaling Technologies, Danvers, MA, USA, 1/400), ER (6F11, Leica, 1/400), and PR (SP2, Thermo Fisher, 1/400) at 4 °C overnight, followed by biotinylated IgG secondary antibodies (Vector Labs). Signal detection was performed using ABC Elite (Vector Labs) for 30 min and 3,3’‐diaminobenzidine (DAKO) for 5 min at room temperature.

### 2.9 Western blot analysis

Organoids and tumors were lysed in RIPA buffer (50 mm Tris pH 8.0, 150 mm NaCl, 0.1% SDS, 0.5% deoxycholate, and 1% Triton) supplemented with complete protease inhibitor tablets (Roche, Basel, Switzerland). Lysates were separated by SDS/PAGE on 4‐20% Bis‐Tris pre‐cast gels (Invitrogen) and transferred onto polyvinylidene difluoride membrane (Millipore, Burlington, MA, USA). Membranes were probed with primary antibodies against AXIN1 (C76H11, Cell Signaling Technologies), pan‐AKT (C67E7, Cell Signaling Technologies), p‐AKT (D9E, Cell Signaling Technologies), GSK3α/β (D75D3, Cell Signaling Technologies), pGSK3α/β (9331, Cell Signaling Technologies), S6 (5G10, Cell Signaling Technologies), pS6 (2211, Cell Signaling Technologies), PRKARIA (20/PKA RIα, BD Bioscience), or GAPDH (loading control; 71.1, Sigma). After primary antibody incubation, membranes were probed using HRP‐conjugated goat anti‐rabbit/mouse IgG secondary antibodies and developed in ECL (GE Healthcare Life Sciences). All western blots are representative of *n* ≥ 2 experiments.

### 2.10 RNA‐sequencing analysis of organoids

Total RNA was extracted from mammary organoids for RNA‐seq profiling using Illumina's TruSeq RNA v2 sample preparation protocol (approx. 50 ng RNA as input), with three biological replicates for each. Libraries were sequenced on an Illumina NextSeq 500. Between 63 million and 84 million 75 bp paired‐end reads were generated for each sample. Reads were aligned to the mouse genome mm10 using rsubread version 2.2.2 [29]. Read counts were obtained for Entrez gene Ids using featureCounts and Rsubread’s inbuilt RefSeq annotation. Gene annotation was downloaded from https://ftp.ncbi.nih.gov/gene/data/gene_info. Obsolete Entrez Ids were removed as were mitochondrial genes or genes on unassembled scaffolds. Genes of type ‘rRNA’, ‘pseudo’, ‘unknown’, or ‘other’ were also filtered. Library sizes were normalized using the TMM method [30]. Differential gene expression analysis was conducted using the quasi‐likelihood pipeline of the edgeR package [31]. Genes were considered to be differentially expressed if they achieved a false discovery rate (FDR) below 5%.

**Fig. 1:**
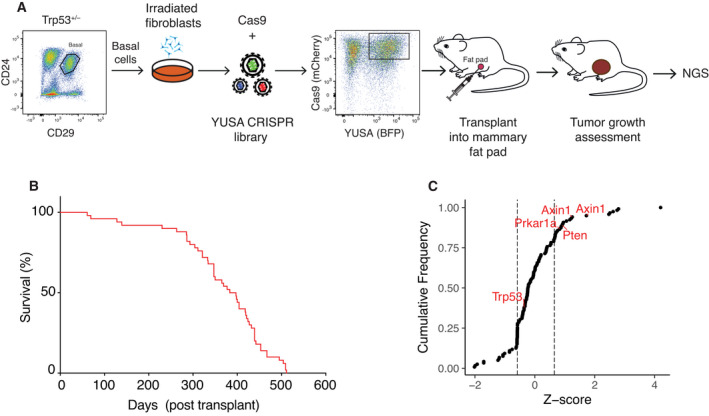
Identification of *Prkar1a* and *Axin1* in a genome‐wide CRISPR/Cas9 screen in *Trp53^+/–^* mice. (A) Schematic diagram of the *in vivo* genome‐wide CRISPR/Cas9 screen in *Trp53^+/–^* mice. BALB/c‐*Trp53^+/–^* basal cells were expanded on a fibroblast feeder layer, then infected with the YUSA‐sgRNA‐library and Cas9 lentiviruses. YUSA‐sgRNA‐library/Cas9 double‐positive cells were sorted and transplanted into the fat pads of BALB/c recipient mice. Tumor development was monitored, and then, arising tumors were subjected to next‐generation sequencing (NGS) for sgRNA alterations. (B) Kaplan–Meier survival curve of BALB/c mice bearing tumors following transplantation with YUSA library edited *Trp53^+/–^* basal cells. Median tumor latency was 373 days (*n* = 79 mice in 3 experiments). (C) Cumulative frequency of genes identified in the CRISPR/Cas9 screen in *Trp53^+/–^* mice (Z‐score normalized, dotted lines indicate 20% mark).

**Fig. 2:**
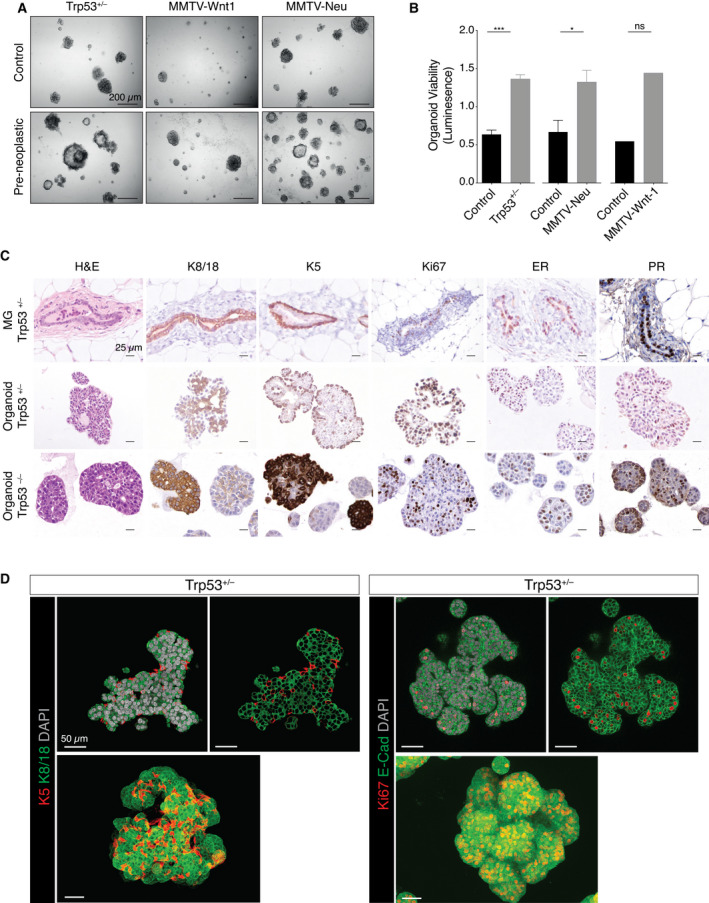
Preneoplastic *Trp53^+/–^* mammary organoids mimic features of *Trp53^+/–^* mammary glands. (A) Representative brightfield images of organoids established from freshly sorted basal cells from *Trp53^+/–^* (*n* = 5), MMTV‐Wnt1 (*n* = 5), and MMTV‐Neu (*n* = 3) mice compared to age‐matched wild‐type organoids after 7 days. Preneoplastic glands were isolated from mice at 4‐5, 2, and 4–5 months for *Trp53^+/–^*, MMTV‐Wnt1, and MMTV‐Neu, respectively. Scale bar, 200 µm. (B) Organoid viability measured by CellTiter Glo Luminescent (Promega) for organoids derived from sorted basal cells isolated from *Trp53^+/–^* (*n* = 3), MMTV‐Wnt1 (*n* = 2), and MMTV‐Neu (*n* = 3) mice compared to organoids from age‐matched wild‐type mice. Error bars represent mean ± s.e.m. ***P < 0.001; *P < 0.05; ns, not significant, two‐sided unpaired Student’s *t*‐test. (C) Immunostaining for K8/18, K5, Ki67, ER, and PR of *Trp53^+/–^* and *Trp53^–/–^* organoids compared to mammary gland sections from *Trp53^+/–^* mice (*n* = 3). Scale bar, 25 µm. (D) Whole‐mount 3‐dimensional confocal images (bottom) and optical sections (top) of preneoplastic mammary organoids derived from *Trp53^+/–^* basal cells stained for K5 and K8/18. Scale bar, 50 µm.

### 2.11 Statistical analysis

**Fig. 3:**
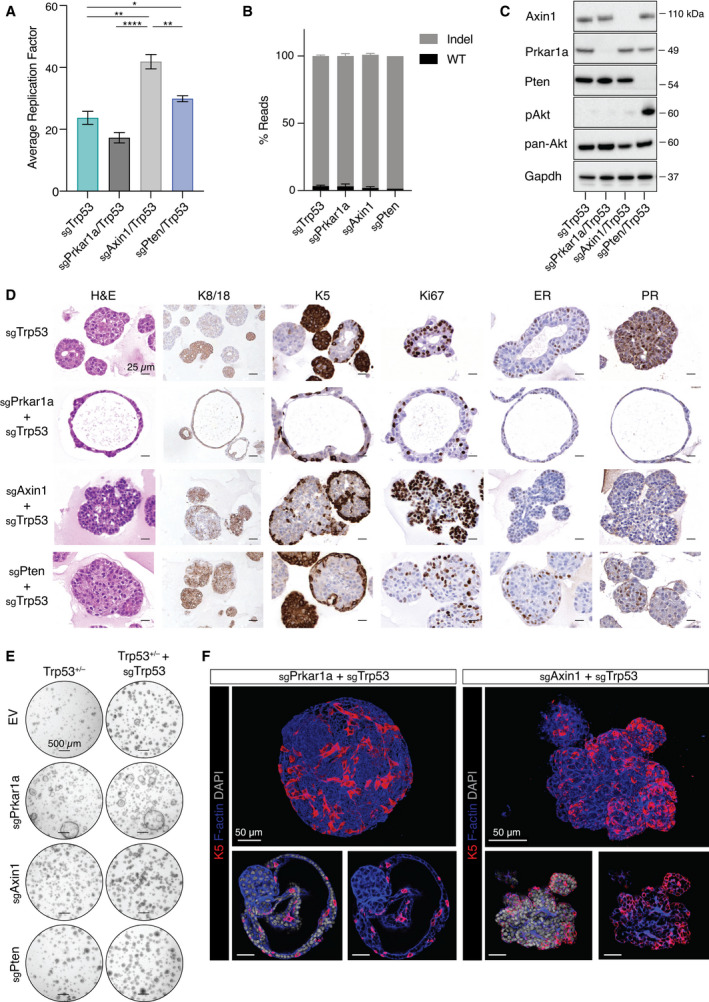
Unique features of *Prkar1a‐* and *Axin1*‐mutated organoids. (A) 5‐week averaged replication factor for *Trp53^+/–^* organoids that were CRISPR/Cas9‐edited for *Trp53*, *Prkar1a*, *Axin1*, or *Pten* (*n* = 3). Error bars represent mean ± s.e.m. *P < 0.05; **P < 0.01; ****P < 0.0001, two‐sided unpaired Student’s *t*‐test. (B) Indel frequency in *Trp53^+/–^* organoids that were CRISPR/Cas9‐edited for *Trp53* (*n* = 4), *Prkar1a/Trp53* (*n* = 3), *Axin1/Trp53* (*n* = 3), or *Pten/Trp53* (*n* = 2). Error bars represent mean ± s.e.m. (C) Western blot analysis of *Trp53^+/–^* organoids following CRISPR‐Cas9 editing of *Trp53*, *Prkar1a*, *Axin1*, or *Pten* for Axin1, Prkar1a, Pten, pAkt, and pan‐Akt expression. Probing for Gapdh provided the loading control (*n* = 3). (D) Immunostaining of *Trp53^+/–^* organoids edited for *Trp53*, *Prkar1a/Trp53*, *Axin1/Trp53*, or *Pten/Trp53* for K8/18, K5, Ki67, ER, and PR expression (*n* = 3). Scale bar, 25 µm. (E) Representative brightfield images of *Trp53^+/–^* organoids edited for the indicated combinations of *Trp53*, *Prkar1a*, *Axin1*, and *Pten* after 1 week culture (*n* = 3). Scale bar, 500 µm. (F) Whole‐mount 3D confocal images (top) and optical sections (bottom) of *Trp53^+/–^* organoids edited for *Prkar1a/Trp53* (left) and *Axin1/Trp53* (right), and stained for K5, F‐actin and DAPI (*n* = 3). Scale bar, 50 µm.

**Fig. 4:**
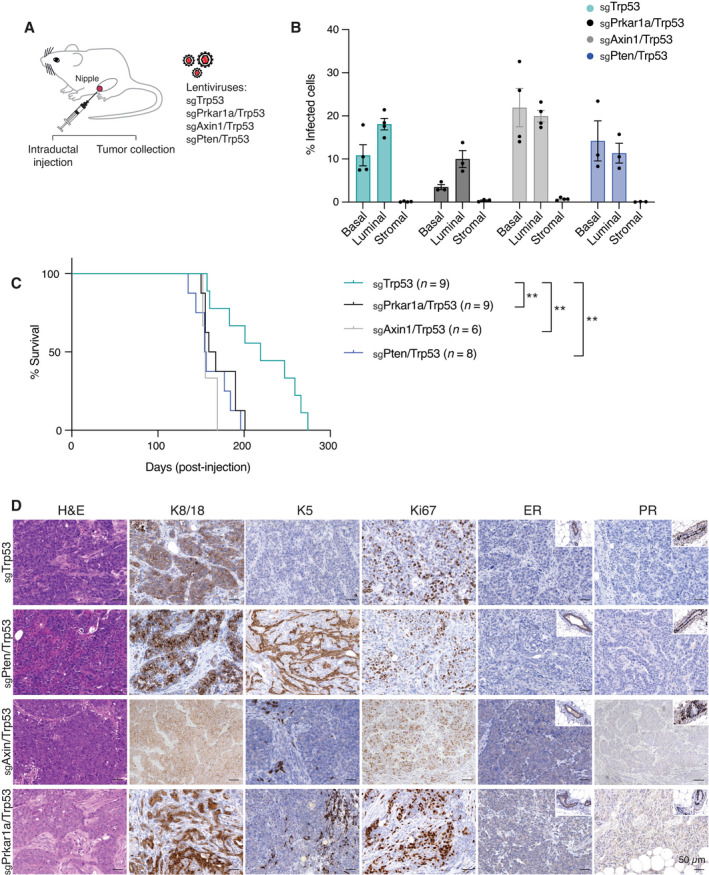
Direct *in vivo* editing of *Prkar1a/Trp53* or *Axin1/Trp53* genes in the ductal epithelium results in mammary tumors. (A) Schematic overview of dual sgRNA‐expressing lentiviral vectors used for intraductal mammary injections. (B) Transduction efficiency of basal, luminal, and stromal cells 2 weeks of postintraductal injection of 10^6^‐10^7^ units (*n* = 3 for *Prkar1a/Trp53* and *Pten/Trp53; n* = 4 for *Axin1/Trp53* and *Trp53*). Error bars represent mean ± s.e.m. (C) Kaplan–Meier plot of survival curves for MMTV‐cre/Cas9 mice‐bearing tumors following intraductal injections with lentiviruses expressing sgRNAs targeting *Trp53* (*n* = 9), *Prkar1a/Trp53* (*n* = 9), *Axin1/Trp53* (*n* = 6), and *Pten/Trp53* (*n* = 8). **P < 0.01. Log‐rank (Mantel–Cox) test. (D) Histological assessment of the expression of K8/18, K5, Ki67, ER, and PR in tumors arising following intraductal injection of lentiviruses expressing sgRNAs targeting *Trp53*, *Prkar1a/Trp53*, *Axin1/Trp53*, or *Pten/Trp53* (*n* = 3). Scale bar, 50 µm.

graphpad Prism software was used to generate Kaplan–Meier survival curves (Figs 1B, 4C and S1A) and other graphs (Figs 2B, 3A,B, 4B, S1B, S2B, S3A–C, and S5C). Error bars in all panels represent ± standard error of the mean (s.e.m.). Statistical analysis was performed using a two‐sided unpaired Students *t*‐test (Figs 2B, 3A and S3A). R studio was used to generate Figs 1C and S4A–D,F–H.

## 3 Results

### 3.1 An *in vivo* genome‐wide screen in *Trp53^+/–^* mice identifies candidate tumor suppressor genes in the mammary gland

To identify tumor suppressors that collaborate with loss of *Trp53*, we conducted an *in vivo* CRISPR/Cas9 screen using a genome‐wide short‐guide RNA (sgRNA) library targeting 19 150 different mouse protein‐coding genes encompassing 87 897 sgRNAs [17] (Fig. 1A). Cells from preneoplastic BALB/c‐*Trp53^+/–^* mammary glands were fractionated by flow cytometry to isolate the CD29^hi^CD24^+^ (basal cell) population, which is enriched for mammary repopulating units (Fig. 1A) [25, 32]. Basal cells, plated on an irradiated fibroblast feeder layer, were co‐transduced with Cas9/mCherry and sgRNA/BFP library lentiviruses, cultured for 7 days, then sorted to collect double‐positive fluorescent epithelial cells for transplantation into the cleared fat pads of recipient BALB/c mice. These were then monitored for tumor development (Fig. 1A).

Tumor latency was found to be variable, ranging from 61 to 505 days (Figs 1B and S1A), presumably reflecting the precise stage of preneoplasia of the transduced cells as well as the influence of the specific sgRNAs. This latency is consistent with the onset of spontaneous tumors previously reported in BALB/c‐*Trp53^+/–^* mice, which varied between 282 and 464 days [10]. Sequencing of tumors revealed enrichment of sgRNAs targeting the well‐known tumor suppressor genes *Trp53*, *Pten*, *Rb1*, and *Nf1* [3, 4, 7]. Manual curation of other enriched sgRNAs identified *Axin1*, *Prkar1a*, *Runx1*, *Tgfbi*, *Tiprl*, *Mafb*, *Pthr2*, *Ggt1*, *Smad3*, *Runx1t1*, and *Pax6* as potential hits of interest (Figs 1C and S1B).

### 3.2 Generation of primary mammary organoids from preneoplastic mouse models

We next examined whether normal mouse mammary organoids could provide a useful tool to study the impact of sequential mutations identified in the screen on neoplastic transformation. Previously, we described the generation of mouse mammary organoids (comprising both the basal and luminal lineages) from single basal cells [26], and normal human breast organoids to model sequential mutagenesis during oncogenesis [33]. Preneoplastic mammary organoids were established from sorted basal cells isolated from three genetically engineered mouse models of breast cancer during the preneoplastic period: BALB/c‐*Trp53^+/–^*, MMTV‐Wnt‐1, or MMTV‐Neu (Fig. 2A). For all models, preneoplastic organoids exhibited higher viability than organoids derived from age‐matched wild‐type mice (Fig. 2A,B). Although organoid passaging was not sustained beyond six passages for these models, long‐term culture was achieved for organoids derived from BALB/c‐*Trp53^–/–^* mammary glands (Fig. S2A,B). Analysis of early passage organoids revealed similar morphology and cytokeratin expression, irrespective of their *Trp53* allele status (Fig. 2C,D). Preneoplastic organoids comprised cuboidal cytokeratin 8/18 (K8/18)‐expressing luminal epithelial cells and cytokeratin 5 (K5)‐expressing basal/myoepithelial cells. Three‐dimensional confocal imaging further revealed that K5^+^ basal cells surround K8/18^+^ luminal cells and highlighted the more elongated shape of myoepithelial cells (Fig. 2D). Moreover, numerous proliferative (Ki67^+^) luminal cells were observed as well as a subset of double‐positive cells that expressed both lineage markers, suggesting deregulation of cell‐fate decisions in the preneoplastic phase (Fig. 2D) compared with normal mammary organoids [26]. Notably, estrogen (ER)‐ and progesterone receptor (PR)‐positive luminal cells were detected in both *Trp53* ^+/–^ and *Trp53* ^–/–^ organoids (Fig. 2C). Together, these findings suggest that short‐term organoid cultures may serve as a useful platform for evaluating the impact of sequential mutations on mammary epithelium.

### 3.3 Deletion of *Axin1* or *Prkar1a* combined with *Trp53* results in mammary organoids exhibiting distinct morphologies

To validate the top candidates identified in the *in vivo* sgRNA screen, we established a mini‐screen based on genetically edited BALB/c‐*Trp53^+/–^* preneoplastic mammary organoids for the testing of 15 candidates. In short‐term assays, CRISPR/Cas9‐based editing of *Prkar1a*, *Axin1*, *Pten*, or *Trp53* in *Trp53* ^+/–^ mammary organoids resulted in significantly higher cell viability, relative to BALB/c‐*Trp53^+/–^* cells infected with the empty vector (Fig. S3A). No change in cell viability was observed in organoids edited with guides targeting *Runx1*, *Tgfbi*, *Tiprl*, *Mafb*, *Nf1*, *Ggt1*, *Smad3*, or *Runx1t1*, although an increase was seen for *Pax6* (Fig. S3B,C). These findings may reflect stochastic enrichment of sgRNAs rather than a direct contribution to tumorigenesis. Alternatively, complex interactions that promote tumorigenesis *in vivo* may not be detected in the organoid assay. For further analysis, we selected two candidate genes, *Axin1* and *Prkar1a*, in addition to the canonical tumor suppressor *Pten*. The scaffold protein Axin1 was originally described as a negative regulator of the Wnt‐signaling pathway [34, 35], although its role in mammary epithelial cells remains unclear. Haplo‐insufficiency of *Prkar1a*, which encodes a regulatory protein in the protein kinase A (PKA) complex, has previously been shown to accelerate sarcoma and thyroid tumor as well as pituitary tumor onset in *Trp53^+/–^* and *Rb^+/–^* mice, respectively [36]. Pertinently, deletion of *Prkar1a* alone in mouse mammary glands led to mammary tumors, albeit with a latency of ~ 12 months [37].

Proliferation changed significantly after genetic editing of *Trp53* ^+/–^ mammary organoids. *Pten*/*Trp53* and *Axin1*/*Trp53* double mutants showed enhanced proliferation, whereas the doubling time of *Prkar1a*/*Trp53‐*edited organoids was comparable to that of *Trp53* mutant organoids (Fig. 3A). Mutagenesis of *Axin1*, *Prkar1a*, *Pten*, or *Trp53* in CRISPR/Cas9‐edited *Trp53* ^+/–^ organoids was confirmed via the identification of indels in more than 90% of reads (Fig. 3B). The high efficiency of editing resulted in reduced protein levels of Prkar1a, Axin1 and Pten (Fig. 3C). As expected, phosphorylation of Akt and its downstream target ribosomal protein S6 was increased in *Pten*‐edited organoids (Figs 3C and S3D). Phosphorylation of S6 appeared to be independent of Akt signaling in *Axin1*‐mutated organoids, suggesting that S6 can be activated by other means (Fig. S3D). Although Gsk3 can bind to Axin1 under normal conditions, no substantial change in active Gsk3 was observed in *Axin1*‐deficient organoids.

Histological assessment and 3D confocal imaging of mammary organoids revealed a striking morphological change in *Prkar1a*/*Trp53‐*edited organoids, which was characterized by an acinar appearance in contrast to the densely packed structure of *Axin1‐* or *Pten‐*edited organoids (Figs 3D‐F and S3E). *Axin1*/*Trp53‐*edited organoids also showed increased budding (Figs 3D‐F and S3E), similar to that observed for normal mouse mammary organoids in the presence of FGF2 [26]. Robust ER and PR expression was only observed in luminal cells of *Pten*/*Trp53* and *Trp53* mutant organoids (Fig. 3D).

To explore the potential molecular basis underlying the morphological differences, we performed RNA‐seq analysis on *Axin1*/*Trp53* and *Prkar1a*/*Trp53‐*edited mammary organoids and compared them to those targeted by *Trp53‐*only guides. Gene signature analysis of *Axin1*/*Trp53* and *Prkar1a*/*Trp53* mutant organoids revealed that the expression signature of *Axin1*/*Trp53* mammary organoids was more aligned with the two luminal populations than the basal/myoepithelial lineage, whereas the converse was true for *Prkar1a*/*Trp53* organoids (Fig. S4A,B). *Axin1*/*Trp53* mammary organoids showed significant downregulation of *Esr1*, consistent with the absence of ER and PR expression in organoids (Fig. S4C). Surprisingly, both agonists (e.g., *Wnt4*, *Wnt10a*) and antagonists (e.g., *Sfrp1*, *Notum*) of Wnt signaling were downregulated in *Axin1*/*Trp53*‐edited organoids (Fig. S4C,D). Notably, β‐catenin localization did not change in *Axin1*/*Trp53*‐edited (nor *Prkar1a*/*Trp53*) organoids, indicating that *Axin1* mutations in mammary organoids do not result in potent activation of canonical Wnt signaling (Fig. S4E). Genes involved in protein catalyzation were increased, possibly for the energy supply necessary for the increased proliferation observed in *Axin1*/*Trp53* mutant organoids (Fig. S4C,D). In parallel, we noted upregulation of phospho‐S6 in these organoids (Fig. S3D). In *Prkar1a*/*Trp53*‐edited organoids, *Snai1* and *Tgfbi* were among the top differentially expressed genes compared to *Trp53*‐edited organoids (Fig. S4F, G). Interestingly, *Tgfbi* was also identified in the *in vivo Trp53^+/–^* screen (Fig. S1B), suggesting that *Tgfbi* may function downstream of PKA signaling and that loss of either can promote mammary tumorigenesis in a mutually exclusive fashion. Analysis of expression in the TCGA confirmed lower expression of *PRKAR1A* in the basal‐like subtype [37] and showed that *AXIN1* levels did not change appreciably across subtypes of breast cancer (Fig. S4H).

### 3.4 Direct *in vivo* genomic editing of *Axin1* or *Prkar1a* with *Trp53* in ductal cells accelerates tumor development

To determine whether *Axin1* and *Prkar1a* could act as *bona fide* tumor suppressor genes, we used an intraductal lentiviral strategy to directly edit these genes in mammary ductal cells *in vivo*. To this end, we engineered lentiviruses expressing dual sgRNAs (*Pten/Trp53*, *Prkar1a/Trp53*, *Axin1/Trp53*) into a single vector and compared these to lentivirus carrying *Trp53* sgRNA alone. Lentiviral transduction units (10^6^–10^7^) were injected intraductally [38] into *MMTV‐cre*‐driven *Rosa28‐LSL‐Cas9‐EGFP* female mice (Fig. 4A). To confirm the efficiency of viral transduction, we performed flow cytometry 2 weeks postinjection. Both basal and luminal epithelial cells were successfully transduced at the following frequencies: 7.4–21.4% for *Trp53* alone, 2.8–13.8% for *Prkar1a/Trp53*, 14–32.6% *Axin1/Trp53*, and 7.8–23.4% for *Pten/Trp53*. Lentiviral infection of stromal fibroblasts was infrequent (Figs 4B and S5A,B).

We next investigated whether mutation of *Axin1* or *Prkar1a* in combination with *Trp53* loss was sufficient to induce mammary tumors. Deletion of *Trp53* alone resulted in tumors with a median onset of 219 days, whereas deletion of two tumor suppressor genes using the dual‐sgRNA lentiviruses accelerated tumor onset by approximately 60 days. Median tumor onset was 163, 155, and 155 days for *Prkar1a/Trp53*, *Axin1/Trp53*, and *Pten/Trp53* mutants, respectively (Fig. 4C). Thus, mutagenesis of *Prkar1a* and *Axin1* (with *Trp53*) exhibited similar oncogenic potency to *Pten/Trp53* mutants in this intraductal model. Indels were observed in tumors from all the different combinations although at reduced frequency compared to mammary organoids (Fig. S5C). Histological examination revealed high‐grade, proliferative carcinomas (as determined by Ki67 and keratin expression), with the majority exhibiting metaplastic features, characterized by the presence of spindle cells and/or squamous differentiation (Fig. 4D). In contrast to the *in vitro Pten/Trp53*‐edited organoids, the expression of ER and PR was negligible in established tumors. These data indicate that hormone receptor expression is downregulated between the preneoplastic state (organoids) and neoplastic progression *in vivo*. Overall, these findings indicate that both *Axin1* and *Prkar1a* loss can augment loss of *Trp53*‐mediated mammary tumorigenesis.

## 4 Discussion

In this report, we describe a pipeline for the identification and validation of mammary tumor suppressor genes using an *in vivo* genome‐wide CRISPR/Cas9 screen combined with genomic editing of ‘preneoplastic’ mammary organoids and direct *in vivo* genetic manipulation of candidate genes in the ductal epithelium. This strategy proved to be valuable for unraveling cooperativity between tumor suppressor genes in mammary tumorigenesis. A number of potential collaborative tumor suppressor genes were identified in the *in vivo* screen, with *Axin1* and *Prkar1a* selected for further investigation. Mutations in either *Axin1* or *Prkar1a* were shown to cooperate with *Trp53*‐deficiency in eliciting mammary tumors, highlighting their potential role as tumor suppressors in the mammary epithelium. In addition to *TP53* and *PTEN* mutations, activating mutations in *PIK3CA* are a common occurrence in breast cancers and *MLL3* has been identified as a *PIK3CA*‐cooperating gene using CRISPR/Cas9 genome editing [16].

Previous studies have implicated abnormalities in *Axin1* and *Prkar1a* expression in breast cancer although their roles in normal development and oncogenesis remain unclear. *Axin1* was among the top 93 driver genes mutated in breast cancer, ranging between 3 and 5% across the different subtypes [6]. In mouse mammary organoids, *Axin1* (and *Trp53*) mutagenesis enhanced organoid proliferation and induced the formation of tumors. These processes occurred in the absence of Wnt pathway activation based on RNA‐seq analysis and β‐catenin localization, thus inferring a Wnt‐independent role for Axin1 despite its apparent association with the β‐catenin destruction complex [34]. Similarly, loss of *Axin1* was found to induce hepatocellular carcinoma (HCC) in the absence of β‐catenin activation [39, 40]. Interestingly, there are two prominent subclasses of HCC, one harboring inactivating mutations in *AXIN1* and the other activating mutations in *CTNNB1*, each of which exhibit very different gene expression programs [41]. Axin1 has also emerged as an important scaffold protein involved in the regulation of signaling pathways unrelated to WNT that include JNK/MAPK [42, 43], mTOR [44], and Trp53 [45].

Recent findings have highlighted a potential role for *Prkar1a* as a tumor suppressor gene. *PRKAR1A* is associated with poor patient prognosis in basal‐like breast cancer [46], and low *PRKAR1A/*high *SRC* expression has been linked to basal‐like and HER2^+^ breast cancers with adverse clinical outcome [37]. Moreover, *Prkar1a* has been shown to act as a tumor suppressor in the mouse mammary gland [37] and to accelerate sarcoma development in *Trp53^+/–^* mice [36]. Using an independent strategy, we identified *Prkar1a* in a genome‐wide screen as a tumor suppressor that collaborates with *Trp53* haplo‐insufficiency and validated its action in mammary oncogenesis by direct *in vivo* genomic editing. While these data suggest that aberrant PKA activity directly contributes to oncogenesis, there are small subsets of breast cancer patients that harbor amplifications in either negative or positive regulators of PKA, suggesting a further layer of complexity [46]. Further studies will be required to define the precise roles of *Axin1* and *Prkar1a* in breast epithelial cells and the mechanisms by which defects in these genes contribute to breast cancer progression.

## 5 Conclusion

Our findings demonstrate the feasibility of performing *in vivo* genome‐wide CRISPR/Cas9 screens using primary mouse mammary epithelial cells to identify collaborating tumor suppressor genes. The validation pipeline that we developed for interrogating candidate genes involved: (a) the genetic engineering of primary mammary organoids *ex vivo* and (b) direct editing of the ductal mammary epithelium *in vivo*.

## Conflict of interest

The authors declare no conflict of interest.

### Peer Review

The peer review history for this article is available at https://publons.com/publon/10.1002/1878‐0261.13179.

## Author contributions

LH, JRW, GJL, and JEV designed the study; JRW, FV, BDC, JFD, CAD, MJGM, ES, MT, and HC performed experiments; YC and GKS performed bioinformatic analyses; MC carried out pathological analysis; MJH and AS provided the CRISPR/Cas9 library and expertise; LH, GJL, and JEV carried out interpretation of data and manuscript writing.

## Supporting information


**Fig. S1.** Identification of potential tumor suppressor genes in a genome‐wide CRISPR/Cas9 screen in *Trp53^+/–^* mice.

**Fig. S2.** Proliferation of *Trp53^+/+^*, *Trp53^+/–^*, and *Trp53* ^–/–^ mammary organoids.

**Fig. S3.** Genetic editing, viability and 3D confocal imaging of organoids.

**Fig. S4.** Gene expression changes in *Axin1/Trp53* and *Prkar1a/Trp53‐*edited organoids.

**Fig. S5.** Direct *in vivo* genetic editing of *Prkar1a/Trp53* and *Axin1/Trp53* genes within the mouse mammary gland.

**Table S1.** Sequences used for mini‐CRISPR/cas9 screen.

## Data Availability

The RNA‐seq data that support the findings of this study have been deposited in the GEO under the accession code GSE184070.
