# Supplementary material for: In vivo genome‐editing screen identifies tumor suppressor genes that cooperate with Trp53 loss during mammary tumorigenesis
Source: Mol Oncol. 2022 Jan 26;16(5):1119–31. doi: 10.1002/1878-0261.13179 (PMC8895454; doi:10.1002/1878-0261.13179)
Supplement: Supplementary file 1 — Fig. S1. Identification of potential tumor suppressor genes in a genome‐wide CRISPR/Cas9 screen in Trp53+/– mice. [file MOL2-16-1119-s006.pdf]

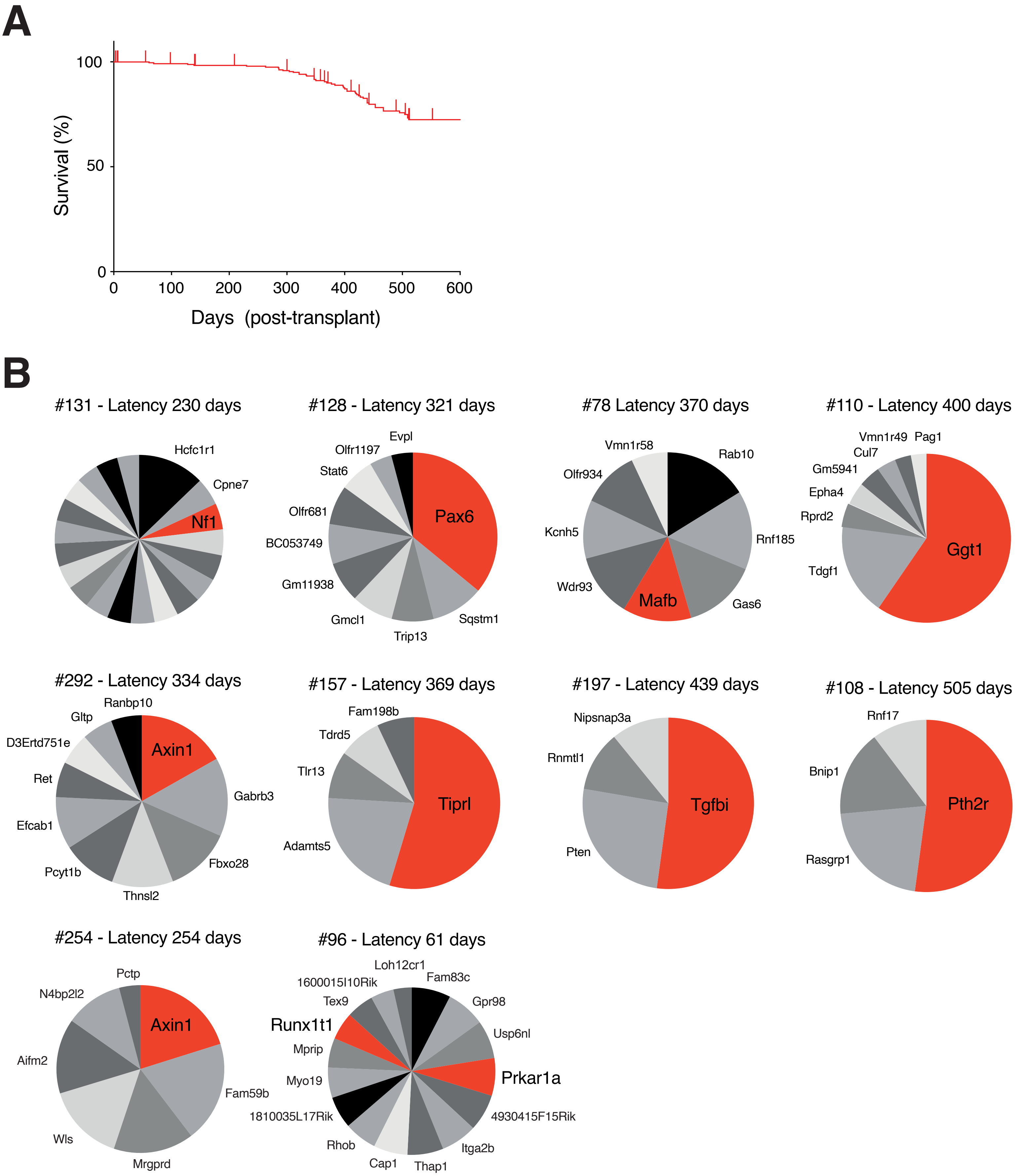

Fig. S1. Identification of potential tumor suppressor genes in a genome-wide CRISPR/Cas9 screen in *$Trp53^{+/-}$* mice. (A) Kaplan-Meier survival curve of BALB/c mice transplanted at 3 - 4 weeks with 2 x 10^4 cells YUSA-edited *$Trp53^{+/-}$* basal epithelial cells (n = 3 experiments, n = 275 mice). Vertical line represents censored events. (B) Distribution of reads per sgRNA in arising tumors, with genes of interest highlighted.
