# Supplementary material for: In vivo genome‐editing screen identifies tumor suppressor genes that cooperate with Trp53 loss during mammary tumorigenesis
Source: Mol Oncol. 2022 Jan 26;16(5):1119–31. doi: 10.1002/1878-0261.13179 (PMC8895454; doi:10.1002/1878-0261.13179)
Supplement: Supplementary file 2 — Fig. S2. Proliferation of Trp53+/+ , Trp53+/– , and Trp53 –/– mammary organoids. [file MOL2-16-1119-s005.pdf]

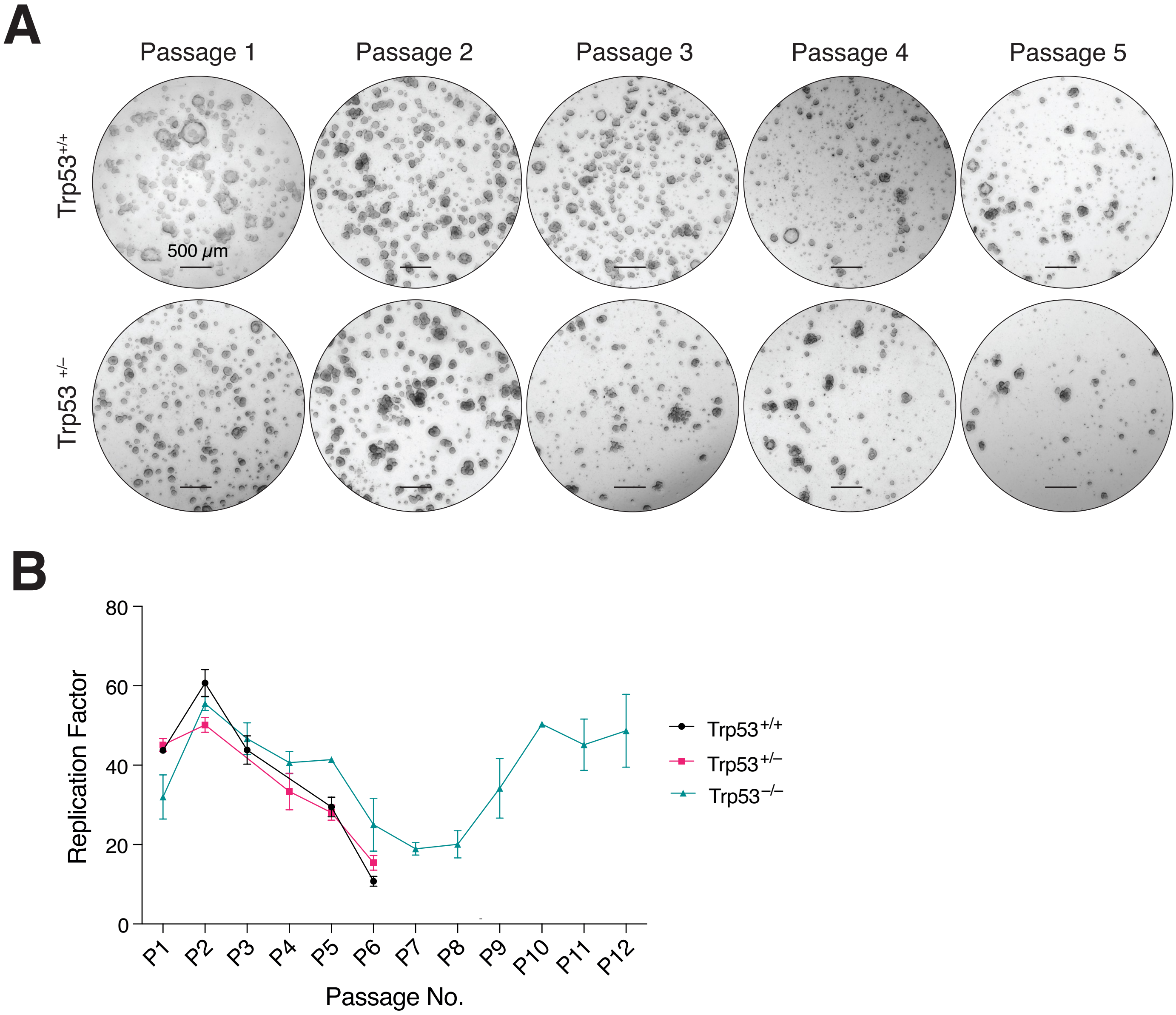

Fig. S2. Proliferation of *Trp53*$^{+/+}$, *Trp53*$^{+/-}$ and *Trp53*$^{-/-}$ mammary organoids. (A) Representative brightfield images of *Trp53*$^{+/+}$ and *Trp53*$^{+/-}$ organoids before each sequential passage (representative of n = 4 experiments). Scale bar, 500 μm. (B) Proliferation/replication factor for *Trp53*$^{+/+}$, *Trp53*$^{+/-}$ and *Trp53*$^{-/-}$ organoids following sequential passaging (n = 2 for *Trp53*$^{-/-}$, n = 4 for *Trp53*$^{+/+}$ and *Trp53*$^{+/-}$). Error bars represent mean ± s.e.m.
