# Supplementary material for: In vivo genome‐editing screen identifies tumor suppressor genes that cooperate with Trp53 loss during mammary tumorigenesis
Source: Mol Oncol. 2022 Jan 26;16(5):1119–31. doi: 10.1002/1878-0261.13179 (PMC8895454; doi:10.1002/1878-0261.13179)
Supplement: Supplementary file 3 — Fig. S3. Genetic editing, viability and 3D confocal imaging of organoids. [file MOL2-16-1119-s001.pdf]

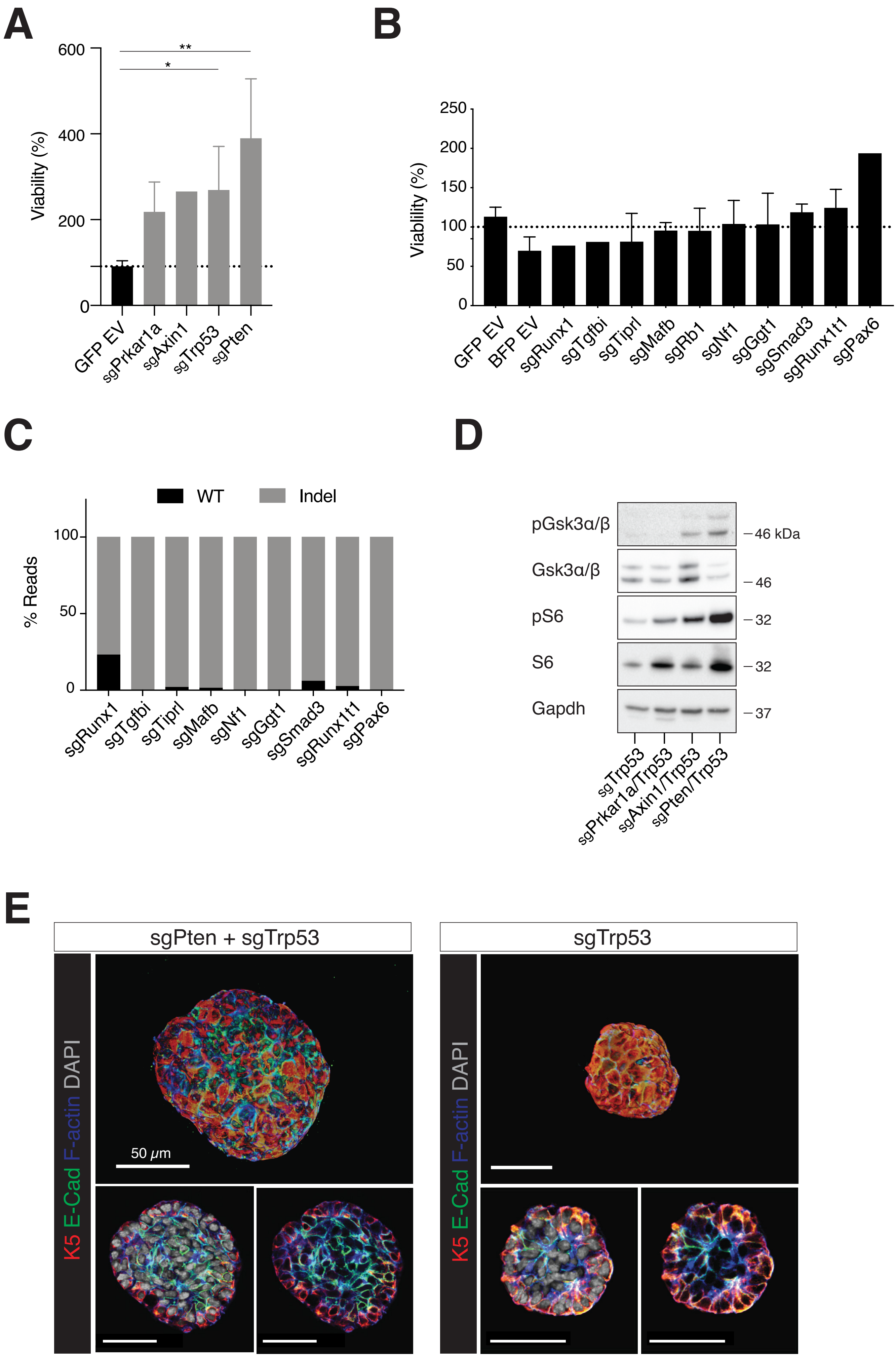
A
600
400
200
0
Viability (%)
**
*
GFP EV
sgPrkar1a
sgAxin1
sgTrp53
sgPten
B
250
200
150
100
50
0
Viability (%)
GFP EV
BFP EV
sgRunx1
sgTgfbi
sgTiprl
sgMafb
sgRb1
sgNf1
sgGgt1
sgSmad3
sgRunx1t1
sgPax6
C
WT
Indel
% Reads
100
50
0
sgRunx1
sgTgfbi
sgTiprl
sgMafb
sgNf1
sgGgt1
sgSmad3
sgRunx1t1
sgPax6
D
pGsk3α/β
Gsk3α/β
pS6
S6
Gapdh
46 kDa
46
32
32
37
sgTrp53
sgPrkar1a/Trp53
sgAxin1/Trp53
sgPten/Trp53
E
sgPten + sgTrp53
sgTrp53
K5 E-Cad F-actin DAPI
50 μm

Fig. S3. Genetic editing, viability and 3D confocal imaging of organoids. (A) Viability of *Trp53*$^{+/-}$ organoids infected with the indicated sgRNA-lentiviruses (n = 4 for GFP-EV, *Trp53* and *Prkar1;* n = 2 for *Axin1*). Error bars represent mean ± s.e.m. * p<0.05; ** p<0.01, two-sided unpaired Student's t-test. (B) Viability of *Trp53*$^{+/-}$ organoids transduced with *Runx1* (n = 2), *Tgfbi* (n = 2), *Tiprl* (n = 3), *Mafb* (n = 4), *Nf1* (n = 4), *Ggt1* (n = 4), *Smad3* (n = 4), *Runx1t1* (n = 3) and *Pax6* (n = 2) sgRNAs. Error bars represent mean ± s.e.m. (C) Indel frequency in *Trp53*$^{+/-}$ organoids that were CRISPR/Cas9-edited for *Runx1*, *Tgfbi*, *Tiprl*, *Mafb*, *Nf1*, *Ggt1*, *Smad3*, *Runx1t1* and *Pax6* (n = 2 except *Ggt1* and *Pax6*, where n = 1). (D) Western blot analysis of *Trp53*$^{+/-}$ organoids for pGSK3α/β, GSK3α, pS6 and S6 expression following CRISPR/Cas9 editing for *Trp53*, *Prkar1a/Trp53*, *Axin1/Trp53* or *Pten/Trp53.* Probing for Gapdh provided the loading control (n = 2). (E) Whole-mount 3D confocal images (top) optical sections (bottom) of *Trp53*$^{+/-}$ organoids edited for *Pten/Trp53* or *Trp53* stained for K5, E-cadherin, F-actin and DAPI (n = 2 per genotype). Scale bar, 50 μm.
