# Supplementary material for: In vivo genome‐editing screen identifies tumor suppressor genes that cooperate with Trp53 loss during mammary tumorigenesis
Source: Mol Oncol. 2022 Jan 26;16(5):1119–31. doi: 10.1002/1878-0261.13179 (PMC8895454; doi:10.1002/1878-0261.13179)
Supplement: Supplementary file 4 — Fig. S4. Gene expression changes in Axin1/Trp53 and Prkar1a/Trp53‐edited organoids. [file MOL2-16-1119-s004.pdf]

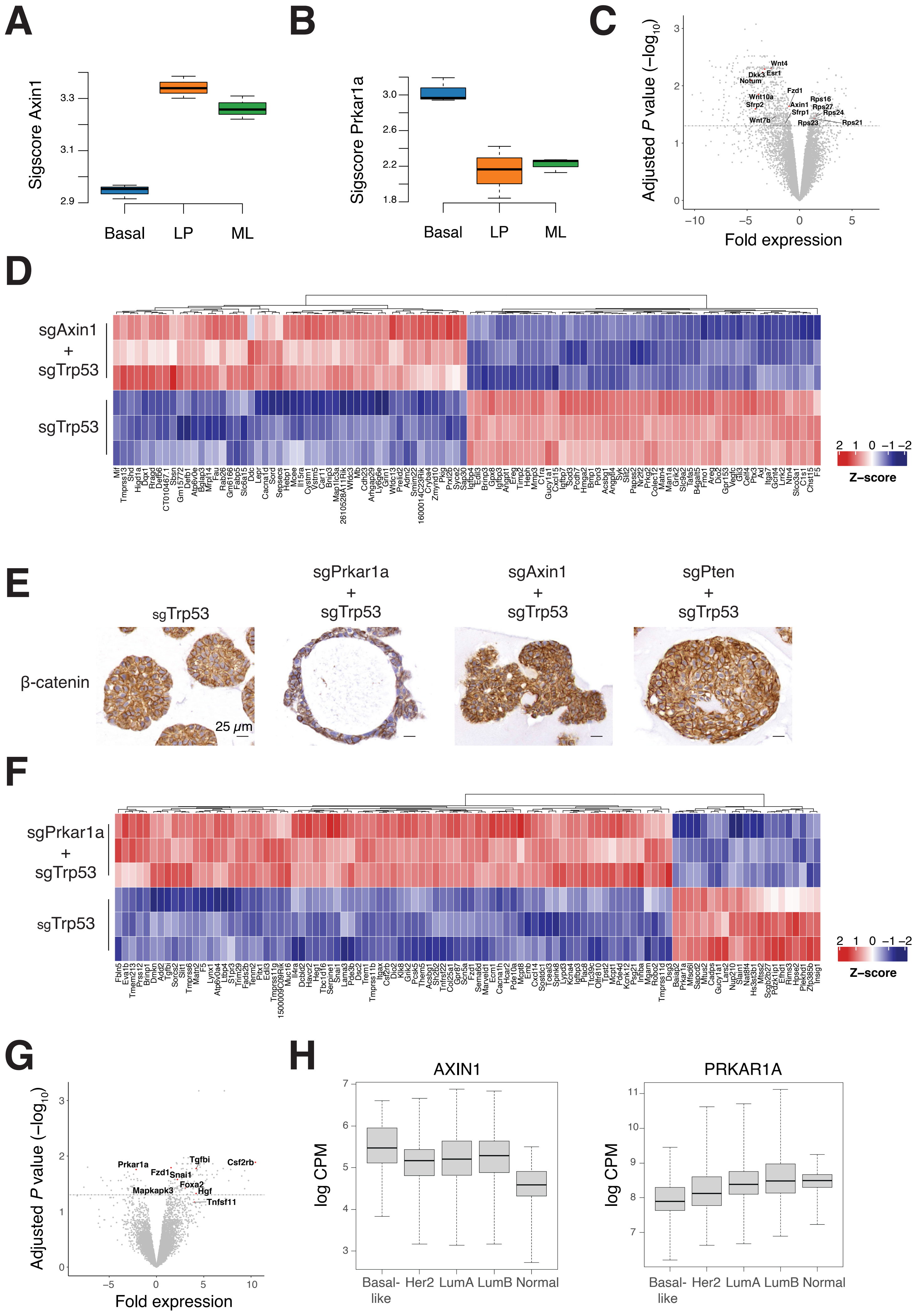
A
Sigscore Axin1
Basal
LP
ML
B
Sigscore Prkar1a
Basal
LP
ML
C
Adjusted P value (−log10)
Fold expression
Wnt4
Dkk3
Esr1
Notum
Fzd1
Wnt10a
Sfrp2
Axin1
Sfrp1
Rps16
Rps27
Rps24
Wnt7b
Rps23
Rps21
D
sgAxin1
+
sgTrp53
sgTrp53
Z–score
E
sgTrp53
sgPrkar1a
+
sgTrp53
sgAxin1
+
sgTrp53
sgPten
+
sgTrp53
β-catenin
25 μm
F
sgPrkar1a
+
sgTrp53
sgTrp53
Z–score
G
Adjusted P value (−log10)
Fold expression
Prkar1a
Tgfbi
Csf2rb
Fzd1
Snai1
Foxa2
Mapkapk3
Hgf
Tnfsf11
H
AXIN1
PRKAR1A
log CPM
Basal-like
Her2
LumA
LumB
Normal

Fig S4. Gene expression changes in *Axin1/Trp53* and *Prkar1a/Trp53*-edited organoids. (A) Boxplot showing the relationship between the gene expression signatures of *Axin1/Trp53* mutant organoids and the normal mouse basal, luminal progenitor (LP) and mature luminal (ML) cell populations [15] (n = 3 per condition). (B) Boxplot showing the relationship between the gene expression signatures of *Prkar1a/Trp53* mutant organoids versus the normal basal, LP and ML populations [15] (n = 3 per condition). (C) Volcano plot for *Trp53 vs Axin1/Trp53* CRISPR/Cas9-edited organoids indicating the down-regulation of multiple Wnt pathway genes and *Esr1,* and the upregulation of multiple ribosome-related genes (n = 3 per condition). (D) Top 50 downregulated DE genes and top 50 upregulated DE genes for *Trp53 vs Axin1/Trp53* CRISPR/Cas9-edited organoids (n = 3 per genotype). (E) Immunostaining of *Trp53*$^{+/-}$ organoids edited for *Trp53*, *Prkar1a/Trp53*, *Axin1/Trp53* or *Pten/Trp53* for β-catenin expression (n = 2 per genotype). Scale bar, 25 *μ*m. (F) Top 100 DE genes for *Trp53 vs Prkar1a/Trp53* CRISPR/Cas9-edited organoids (n = 3 per condition). (G) Volcano-plot for *Trp53 vs Prkar1a/Trp53* CRISPR/Cas9-edited organoids, highlighting the up- or down-regulated DE genes of interest (n = 3 per genotype). (H) Box plots showing expression of *AXIN1* and *PRKAR1A* according to breast cancer subtype from the TCGA. Vertical axis shows average expression as log2 counts per million. Box plots show quartiles, minimum and maximum.
