# Supplementary material for: In vivo genome‐editing screen identifies tumor suppressor genes that cooperate with Trp53 loss during mammary tumorigenesis
Source: Mol Oncol. 2022 Jan 26;16(5):1119–31. doi: 10.1002/1878-0261.13179 (PMC8895454; doi:10.1002/1878-0261.13179)
Supplement: Supplementary file 5 — Fig. S5. Direct in vivo genetic editing of Prkar1a/Trp53 and Axin1/Trp53 genes within the mouse mammary gland. [file MOL2-16-1119-s002.pdf]

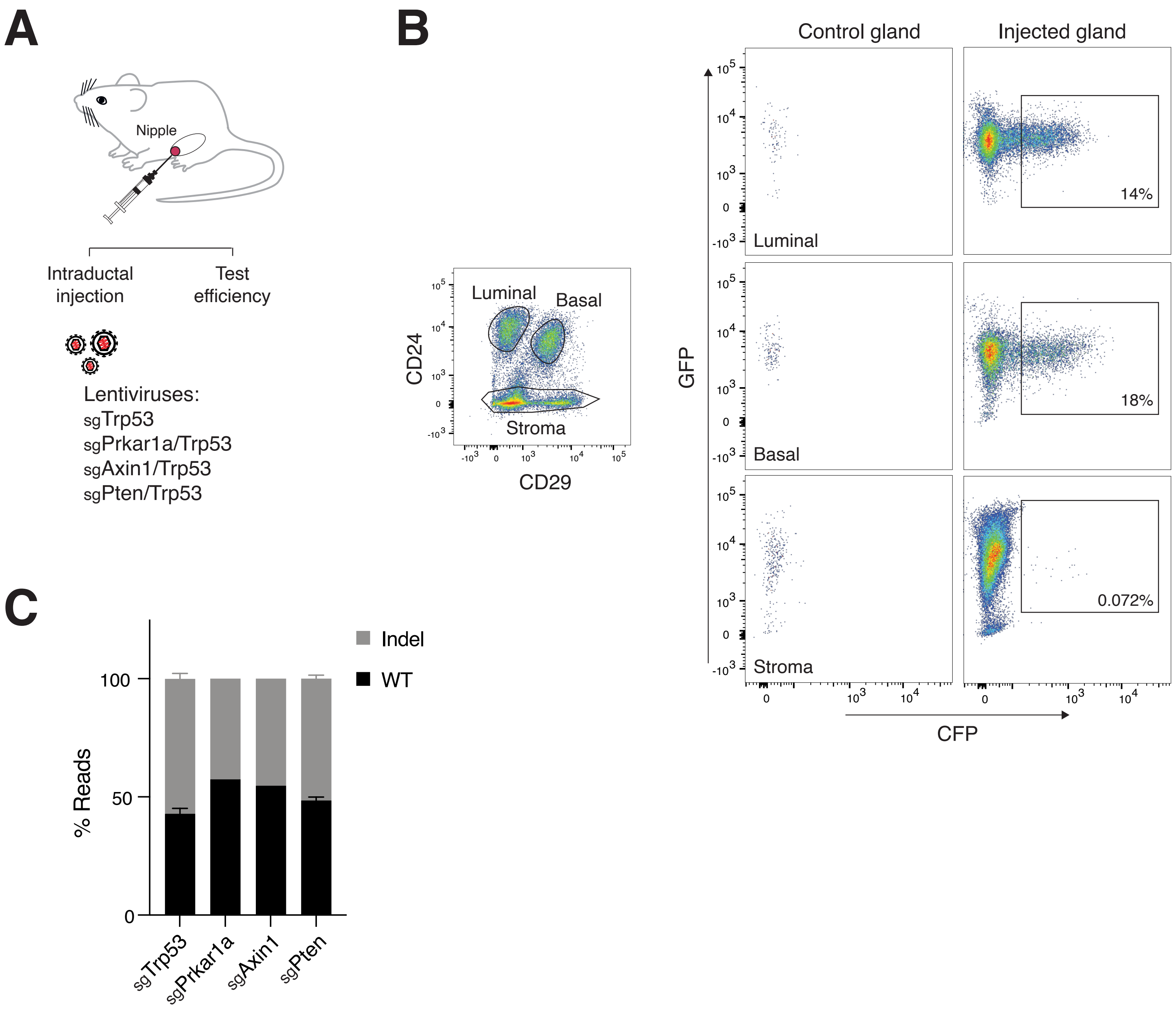

Fig. S5. Direct *in vivo* genetic editing of *Prkar1a/Trp53* and *Axin1/Trp53* genes within the mouse mammary gland. (A) Schematic overview of the dual sgRNA-expressing lentiviral vectors used for intraductal injection into the fourth mammary gland. (B) Gating strategy showing the transduction efficiency for the basal, luminal and stromal populations at two weeks post-intraductal injection. (C) Indel frequency in *Trp53*$^{+/-}$ bulk tumors edited for *Trp53* (n = 4), *Prkar1a/Trp53* (n = 2), *Axin1/Trp53* (n = 2) or *Pten/Trp53* (n = 4). Error bars represent mean ± s.e.m.
