# Supplementary material for: In vivo genome‐editing screen identifies tumor suppressor genes that cooperate with Trp53 loss during mammary tumorigenesis
Source: Mol Oncol. 2022 Jan 26;16(5):1119–31. doi: 10.1002/1878-0261.13179 (PMC8895454; doi:10.1002/1878-0261.13179)
Supplement: Supplementary file 6 — Table S1. Sequences used for mini‐CRISPR/cas9 screen. [file MOL2-16-1119-s003.pdf]

**Table S1.** Sequences used for mini-CRISPR/cas9 screen.

| Gene | | sgRNA sequence |
|---|---|---|
| *Trp53* | Exon 5 | GAGCGCTGCTCCGATGGTGA |
| *Prkar1a* | Exon 3 | CCCAATCCAGTGGTGAAGGG |
| *Prkar1a* | Exon 3 | CGTCCTCCCTCGAGTCAGTA |
| *Axin1* | Exon 2 | TCAAGTAGACGGTACAACGA |
| *Axin1* | Exon 3 | GTTTCACCGAAGATGCCCCC |
| *Pten* | Exon 1 | AAACAAAAGGAGATATCAAG |
| *Pax6* | Exon 9 | GGCAAACACATCTGGATAAT |
| *Pax6* | Exon 10 | AATGTGACTAGGAGTGTTGC |
| *Smad3* | Exon 2 | GCTCCATGGCCCGTAATTCA |
| *Smad3* | Exon 2 | ACGTTATCTACTGCCGCCTG |
| *Tgfbi* | Exon 3 | CCTGGGACCTTTTCATATCC |
| *Tgfbi* | Exon 4 | TATGAGACCATGGGAGTTGT |
| *Mafb* | Exon 1 | GAAGAAGGCACGGAGCTGCA |
| *Mafb* | Exon 1 | GGTTCAGTCGGACTGAAGCT |
| *Ggt1* | Exon 3 | CATACACAGCAGGCTTGCGA |
| *Ggt1* | Exon 3 | CCTACAGGAAGGTGGTTCCG |
| *Runx1t1* | Exon 7 | CGACCATGCACTATTAGCCC |
| *Runx1t1* | Exon 7 | GCACTATTAGCCCAGGCCAG |
| *Tiprl* | Exon 2 | TGTTTGGAGACAACGTTCTA |
| *Tiprl* | Exon 2 | TGGAATAGAGTTCAATGCTA |
| *Pth2r* | Exon 3 | TGATTGAAGTCGTAAACATA |
| *Pth2r* | Exon 3 | TGAAGTCGTAAACATACGGA |
| *Runx1* | Exon 4 | GGTCGTTGAATCTCGCTACC |
| *Runx1* | Exon 4 | TAGCGAGATTCAACGACCTC |
